# Validation of Bmi1 as a Therapeutic Target of Hepatocellular Carcinoma in Mice

**DOI:** 10.3390/ijms151120004

**Published:** 2014-11-03

**Authors:** Shibo Qi, Bin Li, Tan Yang, Yong Liu, Shanshan Cao, Xingxing He, Peng Zhang, Lei Li, Chuanrui Xu

**Affiliations:** 1 School of Pharmacy, Tongji Medical College, Huazhong University of Science and Technology, Wuhan 430030, China; E-Mails: qi601960314@gmail.com (S.Q.); tjlibin@hust.edu.cn (B.L.); M201475282@hust.edu.cn (T.Y.); liuxiulankl@gmail.com (Y.L.); M201475285@hust.edu.cn (S.C.); M201475065@hust.edu.cn (L.L.); 2 Department of Hepatology, Tongji Medical College, Huazhong University of Science and Technology, Wuhan 430030, China; E-Mail: xxhe@tjh.tjmu.edu.cn; 3 Department of Oncology of Tongji Hospital, Tongji Medical College, Huazhong University of Science and Technology, Wuhan 430030, China; E-Mail: pengzhang@tjh.tjmu.edu.cn

**Keywords:** Bmi1, HCC, knockdown, proliferation, treatment

## Abstract

Bmi1 is a member of the polycomb group family of proteins, and it drives the carcinogenesis of various cancers and governs the self-renewal of multiple types of stem cells. Our previous studies have revealed that *Bmi1* acts as an oncogene in hepatic carcinogenesis in an *INK4a/ARF* locus independent manner. However, whether Bmi1 can be used as a potential target for hepatocellular carcinoma treatment has not been fully confirmed yet. Here, we show that perturbation of Bmi1 expression by using short hairpin RNA can inhibit the tumorigenicity and tumor growth of hepatocellular carcinoma cells both *in vitro* and *in vivo*. Importantly, *Bmi1* knockdown can block the tumor growth, both in the initiating stages and the fast growing stages. Cellular biology analysis revealed that Bmi1 knockdown induces cell cycle arrest and apoptosis. Our findings verify Bmi1 as a qualified treatment target for hepatocellular carcinoma (HCC) and support Bmi1 targeting treatment with chemotherapeutic agents.

## 1. Introduction

Hepatocellular carcinoma (HCC) is the third leading cause of death from cancers and the fifth most common malignancy worldwide [1]. HCC is often diagnosed at an advanced stage when it is no longer amenable to curative therapies [2]. Because of the lack of therapeutics, mining the potential therapeutic targets is one of the pivotal missions of fundamental research of liver cancers.

In the past few decades, epidemiologic data have confirmed that chronic viral infections and hepatotoxic agents serve as the major risk factors [3,4], but the molecular pathogenesis of HCC is still largely unknown. It is presumed that the initiation and progression of HCC result from cumulative genetic and epigenetic events, similar to those observed in other solid tumors. The most frequently involved tumor suppressor proteins driving hepatic carcinogenesis are pRb, p53, the M6P/IGF2 receptor and E-cadherin [5]. Activation of oncogenes, such as c-Myc, Met and CTNND1, has also been detected in multiple subsets of HCC [6]. Alongside these oncogene alterations, the major pathways implicated in HCC are intensively investigated. These pathways include the RAF/mitogen-activated protein kinase/extracellular signal-regulated kinase (RAF/MEK/ERK) pathway, PI3 kinase/AKT/mammalian target of rapamycin (PI3K/AKT/mTOR) pathway, canonical WNT/β-catenin pathway, insulin-like growth factor pathway, hepatocyte growth factor/*c-MET* pathway and growth factor-regulated angiogenic signaling pathway [7]. Treatment studies targeting these signaling cascades related to cell survival and proliferation are widely conducted in preclinical and early clinical studies [8].

Besides the oncogenes stated above, Bmi1 is another critical oncogene that mediates hepatic carcinogenesis [9]. Bmi1 is a member of the mammalian polycomb group of multimeric transcriptional repressors and is involved in the regulation of development, stem cell self-renewal, cell cycle and senescence [10,11,12,13]. Bmi1 was first identified as an oncogene, because it can cooperate with *c-Myc* to induce murine B-cell lymphoma [14]. Since then, overexpression of Bmi1 has been reported in multiple tumor types, including breast cancer [15], colon carcinoma [16], non-small cell lung cancer [17,18], glioblastoma [19], ovarian cancer [20], bladder cancer [21] and nasopharyngeal carcinoma [22]. Similar to many types of solid tumors and leukemia [23], aberrant expression of Bmi1 is also found in human HCC [24,25]. Chiba and colleagues found that *Bmi1* gene is overexpressed in many HCC cell lines, and knockdown of Bmi1 can reduce the side population in HCC cells [26]. Our previous study also showed that Bmi1 is overexpressed in almost 1/3 of HCC patients, and Bmi1 can cooperate with Ras to induce HCC formation in mice [25]. All of these data support that Bmi1 functions as an oncogene in HCC.

As a major member of the PcG family of proteins, Bmi1 plays important roles during the multiple types of tumorigenesis by epigenetic gene regulation [27]. The molecular mechanisms underlining the functions of Bmi1 in carcinogenesis have been extensively explored. Several studies have revealed that Bmi1 mainly promotes tumor development by repressing INK4a/ARF locus, which can induce cell senescence and inhibit the proliferation of cancer cells [11,13,18]. In HCC, however, Bmi1 was shown to drive HCC pathogenesis independent of repressing INK4a/ARF [24,25]. In addition, the cellular mechanism of how Bmi1 induces HCC and maintains HCC growth is not fully understood. In our past study, no senescence was observed upon Bmi1 repression in HCC [25]. Hence, the exact mechanisms of Bmi1 in HCC carcinogenesis are still elusive.

To validate the feasibility of using Bmi1 as a potential target for HCC treatment, here, we report that knockdown of Bmi1 gene inhibits HCC cell proliferation *in vitro* and *in vivo*, both in the onset periods and the fast growing periods of the neoplasm. The cellular mechanism study revealed that Bmi1 knockdown inhibits HCC cell growth by inducing both cell cycle arrest and apoptosis.

## 2. Results and Discussion

### 2.1. Bmi1 Knockdown Inhibits the Growth of Hep3B Cells by Inducing Cell Cycle Arrest

**Figure 1:**
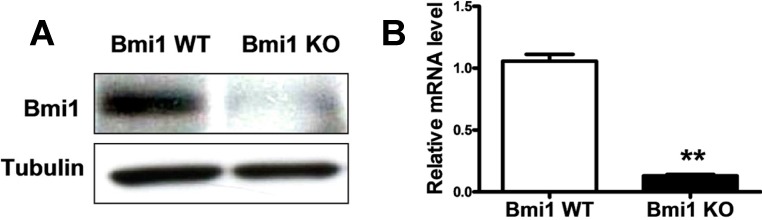

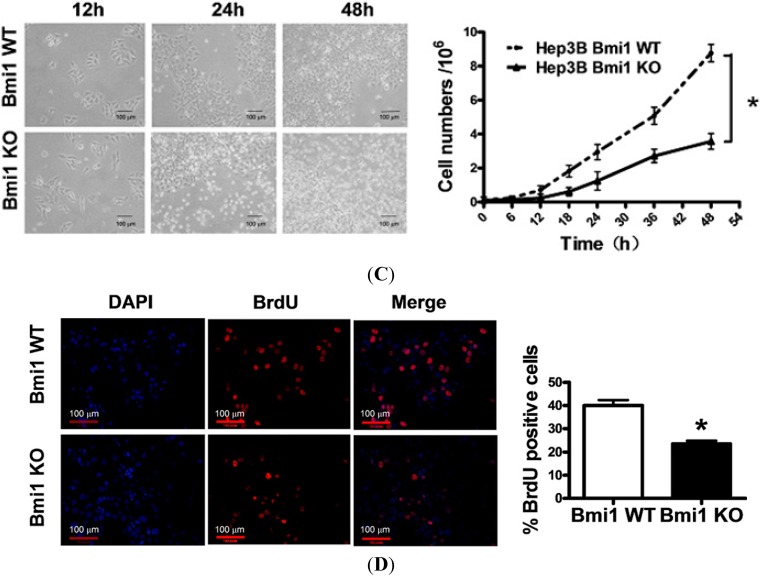
Knocking down Bmi1 inhibits the proliferation of Hep3B cells *in vitro*. Western blotting, proliferation and BrdU incorporation assays were done by using the Hep3B cells after lentivirus infection and puromycin selection for three days. (**A**) Western blotting detection of Bmi1 expression. Tubulin was used as a loading control; (**B**) Real-time qRT-PCR detection of mRNA level of Bmi1 (*n* = 3); (**C**) Cellular morphology of Bmi1 knockdown Hep3B cells. Cells were plated in six-well plates for 1 × 10^5^ cells per well and observed at three time points. Cells were counted after trypan blue staining by using a blood counting chamber (*n* = 3 wells); and (**D**) Proliferation detection of cells by the BrdU incorporation assay. The nucleus was stained blue by DAPI (4',6-diamidino-2-phenylindole), and BrdU stained red. The percentage of BrdU-positive cells was determined by counting BrdU-positive cells and total cells in the same fields (*n* = 3). Data are expressed as the mean ± SD (standard deviation). * *p* < 0.05, and ** *p* < 0.01.

To explore the effects of Bmi1 repression on HCC growth, we first checked the impact of Bmi1 knockdown (KO) on HCC cells *in vitro*. Hep3B cells transfected with Bmi1 shRNA lentivirus or control vector lentivirus were selected by 1 μg/mL of puromycin. Then, the Bmi1 expression and the proliferation of Hep3B cells were examined. Western blotting showed that the expression of Bmi1 was significantly inhibited in Bmi1 KO Hep3B cells (Figure 1A). Quantitative RT-PCR also showed that the *Bmi1* mRNA level decreased to 0.12-fold in Bmi1 KO Hep3B cells (Figure 1B). The phenotypic observation that plenty of Bmi1 KO cells were detached from the culture dish indicated obvious apoptosis or cell death (Figure 1C). Growth curve analysis showed that the growth of Hep3B cells was significantly impaired upon Bmi1 knockdown (Figure 1C). Reduced BrdU staining in Bmi1 KO Hep3B cells confirmed the inhibited proliferation of Bmi1 KO Hep3B cells (Figure 1D). These results clearly indicated that the Bmi1 KO significantly inhibited the growth of HCC cells.

**Figure 2:**
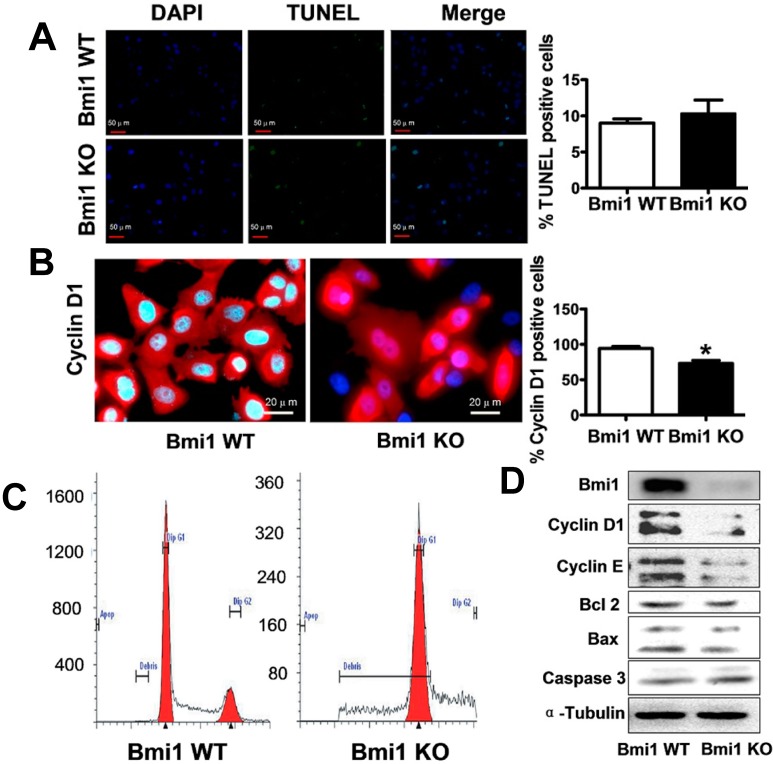
Bmi1 downregulation inhibits Hep3B cell growth by inducing cell cycle arrest. Hep3B cells were infected with Bmi1 shRNA lentivirus and were selected by puromycin. Then, 1 × 10^5^ Hep3B cells were seeded in six-well plates with a cover slip. After 48 h, the cells were processed for western blot, immunofluorescence staining and FACS assays. (**A**) Apoptotic detection by TUNEL assay (100×). The nucleus was stained with blue DAPI, and TUNEL is shown as green; (**B**) The immunofluorescence staining of cell cycle protein cyclin D1 (400×). The nucleus was stained with blue DAPI, and cyline D1 was stained red. Quantitative analyses were conducted by counting staining positive cells and total cells in the same fields (*n* = 3 fields); (**C**) Cell cycle analysis by FACS. For FACS detection, 2 × 10^5^ cells were seeded in six-well plates and cultured for 24 h. A total of 5 × 10^4^ cells were detected for each sample; and (**D**) Western blotting detection of apoptosis and cell cycle-associated proteins. Data are expressed as the mean ± SD. * *p* < 0.05.

We further explored the cellular mechanism of Hep3B cell growth inhibition by Bmi1 knockout. We first performed the TdT-mediated dUTP nick end labeling (TUNEL) assay and found no significantly increased apoptosis in Bmi1 KO Hep3B cells (Figure 2A). Then, we conducted cell cycle analysis through both immunostaining and fluorescence-activated cell sorting (FACS). Immunofluorescence staining showed that the cyclin D1 expression was blocked in some Bmi1 KO Hep3B cells (Figure 2B). Cell cycle analysis by FACS showed that Bmi1 KO Hep3B cells were accumulated in the G1 phase, suggesting a G1 phase arrest in Bmi1 KO cells (Figure 2C). These data collectively suggest that the Bmi1 KO inhibits HCC cell growth mainly by inducing cell cycle arrest. Western blot results showed that expression of cell cycle protein cyclin D1 and cyclin E were downregulated, whereas the expression of apoptosis-associated proteins Bcl-2, Bax and caspase-3 showed no significant changes (Figure 2D). These results suggest that Bmi1 KO in Hep3B cells induced cell cycle arrest in the G1 phase, but did not induce apoptosis *in vitro*.

### 2.2. Bmi1 Knockdown Impairs the Tumor Formation Ability of HCC Cells in Nude Mice

**Figure 3:**
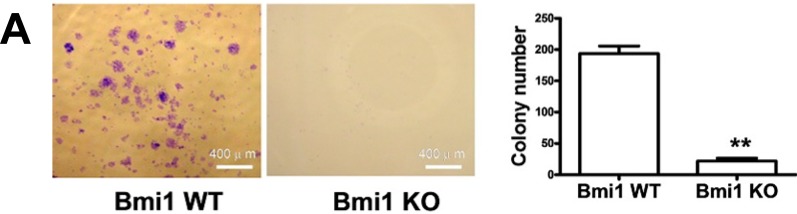

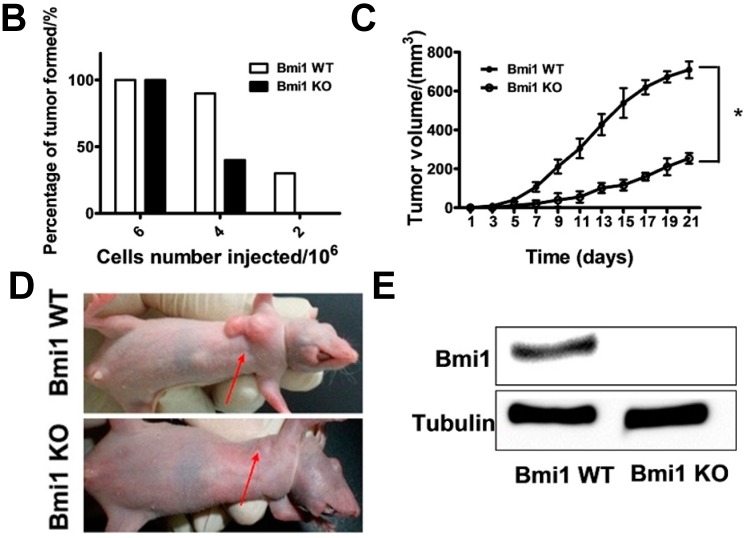
Bmi1 knockdown impairs the tumor formation ability of Hep3B cells in nude mice. (**A**) Colony formation assay of Bmi1 knockdown Hep3B cells. One thousand Hep3B cells were seeded in six-well plates and cultured for three days. For the quantitative analysis, three wells of colonies were counted; (**B**) The tumor-initiating ability of Hep3B cells in nude mice. The tumor formation was observed for two weeks after inoculation (*n* = 10 mice/group); (**C**) Growth curves of the tumors formed by Bmi1 WT and KO Hep3B cell injection. Bmi1 WT or KO Hep3B cells (6 × 10^6^) were injected into nude mice subcutaneously, and the tumor volumes were measured every other day; (**D**) The tumors formed in recipient mice by injecting 6 × 10^6^ Bmi1 KO or WT Hep3B cells. The red arrows in each image are used to indicate the tumors; and (**E**) Bmi1 expression detection by western blotting in the formed tumors. Data are expressed as the mean ± SD. * *p* < 0.05, and ** *p* < 0.01.

To determine the effects of Bmi1 KO on HCC growth *in vivo*, we first checked the impact of Bmi1 KO on the tumorigenicity of Hep3B. The colony formation assay showed that Bmi1 KO impaired the colony formation ability of Hep3B cells significantly. Both the colony numbers and sizes of Bmi1 KO Hep3B cells were significantly lower than those of WT (wild type) Hep3B cells (Figure 3A). To assay the tumorigenicity of Bmi1 KO Hep3B cells in mice, both Bmi1 WT and KO Hep3B cells were inoculated into the BALB/c nude mice with three different cell numbers, 2 × 10^6^, 4 × 10^6^ and 6 × 10^6^ cells (*n* = 10). We found that when 6 × 10^6^ cells were injected, both the WT and Bmi1 KO Hep3B cells could form tumors in the nude mice. When the cell number reduced to 4 × 10^6^, most of the WT cell-injected mice (9/10) formed tumors, whereas less than half of the Bmi1 KO cell-injected mice (4/10) formed tumors. When the cell numbers reduced to 2 × 10^6^, Bmi1 KO Hep3B cells failed to initiate subcutaneous tumors in any recipient mice. However, WT Hep3B cells still formed tumors in three of 10 injected mice (Figure 3B). For the tumors derived from 6 × 10^6^ cell injection, growth curve showed that the tumor formation from the Bmi1 KO cells was significantly delayed compared to the WT cells, and Bmi1 KO significantly repressed tumor growth (Figure 3C). In addition, the sizes of Bmi1 KO tumors were much smaller than those of WT tumors (Figure 3D). The Bmi1 expression in the Bmi1 KO tumors was confirmed by western blotting and showed that the Bmi1 shRNA lentivirus can constantly inhibit the expression of Bmi1 for at least 20 days (Figure 3E). These results collectively illustrate that the tumor-initiating capacity of Hep3B cells is profoundly impaired by Bmi1 knockdown.

### 2.3. Conditional Knockdown of Bmi1 by Doxycycline Inhibits the HCC Cell Growth in Vitro

**Figure 4:**
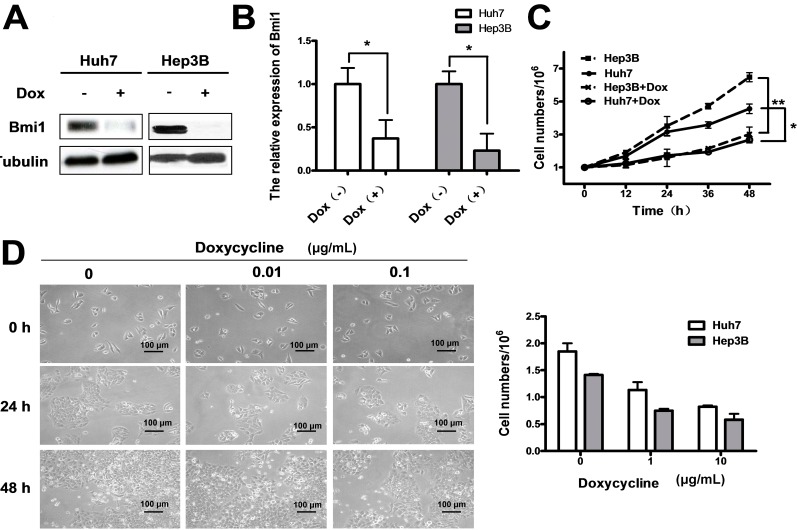
Conditional knockdown of Bmi1 by the tet-on system inhibits the HCC cell growth *in vitro.* Hep3B and Huh7 cells were infected with lentivirus carrying inducible Bmi1 shRNA. After 48 h of puromycin selection, 0.1 μg/mL of doxycycline was added in the culture medium to activate the Bmi1 shRNA expression. After 48 h of doxycycline treatment, the cells were processed for western blot, qRT-PCR and proliferation assays. (**A**) Western blotting of Bmi1 in the Hep3B and Huh7 cells transfected with Bmi1 shRNA lentivirus. Hep3B and Huh7 cells were infected with lentivirus carrying inducible Bmi1 shRNA, and 0.1 μg/mL of doxycycline were added in the culture medium to activate the Bmi1 shRNA expression; (**B**) qRT-PCR results of the Bmi1 mRNA level in Huh7 and Hep3B cells transfected with Bmi1 shRNA by pLKO-Tet-On lentivirus; (**C**) The growth curves of Huh7 and Hep3B cells when the *Bmi1* gene was knocked down. Cells were cultured in medium with 0.1 μg/mL of doxycycline after infecting with lentivirus, and cell numbers were counted at different time points after trypsin digestion; and (**D**) Cell growth of Huh7 and Hep3B cells treated with different concentrations of doxycycline. The morphology of Hep3B cells treated with doxycycline is shown on the left. The Huh7 and Hep3B cell numbers were counted after 1 × 10^5^ cells were cultured in six-well plates for 48 h. Data are expressed as the mean ± SD. * *p* < 0.05, and ** *p* < 0.01.

Although knockdown of Bmi1 significantly impaired the tumorigenicity of HCC cells *in vitro* and in nude mice, the effect of Bmi1 knockout on HCC growth *in vivo* was still unclear. Clinically, treatments only happen after cancer diagnosis, rather than ahead of the tumor formation. Thus, we further asked whether knockdown of Bmi1 can block the growth of HCC in the fast growing stages, at which most cancer patients are diagnosed. To address this question, we cloned the Bmi1 shRNA to an inducible lentivirus expression vector, pLKO-Tet-On plasmid, and prepared the Bmi1/pLKO-Tet-On lentivirus. In these infected cells, doxycycline can trigger the Bmi1 shRNA expression and then silence the Bmi1 expression. The results showed that treatment with 0.1 μg/mL of doxycycline for 24 h significantly inhibited Bmi1 expression in Hep3B and Huh7 cells transfected with Bmi1/pLKO-Tet-On lentivirus (Figure 4A). Quantitative RT-PCR showed that Bmi1 expression was reduced to 30% and 20%, respectively, in Huh7 and Hep3B cells by doxycycline treatment (Figure 4B). Growth curve analysis showed that the proliferation of Huh7 and Hep3B cells was significantly inhibited upon Bmi1 knockdown (Figure 4C). The dose escalation efficacy assay by treating the cells with various concentration of doxycycline showed that doxycycline treatment inhibits the growth of Hep3B and Huh7 cells in a dose-dependent manner. A higher concentration (0.01 *vs.* 0.1 μg/mL) of doxycycline resulted in stronger blocking effects 60% inhibition *vs.* 40% inhibition), and treatments for a longer time enhanced the effects, as well (Figure 4D). These results suggest that Bmi1 KO by doxycycline inhibits the cell growth in a dose-dependent manner *in vitro*.

### 2.4. Shutdown of Bmi1 Represses the Tumor Growth in the Fast Growing Period in Nude Mice

**Figure 5:**
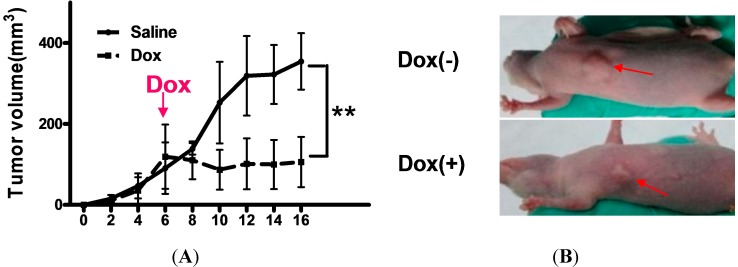

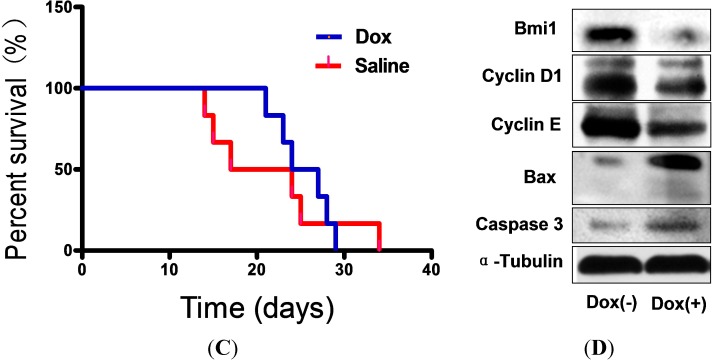
Shutdown of Bmi1 represses the growth of the fast growing tumor in nude mice. (**A**) The growth of tumors in mice with or without doxycycline for 10 days. For the xenograft tumor model, 6 × 10^6^ Hep3B cells transfected with pLK-Tet-on-Bmi1 lentivirus were injected per nude mouse. The tumor volume was measured every other day. The treatment group was supplied with 1.5 mg/mL of doxycycline in the drinking water from the sixth day, when the tumors grew to ~100 mm^3^, whereas the control group was supplied still with regular autoclaved water. The pink arrow indicates that the treatment group begun to be supplied with doxycycline (DOX) in the drinking water; (**B**) The doxycycline-treated mice had smaller-sized tumors compared with control group mice. The pictures were taken on the sixth day after doxycycline treatment. The red arrows in each image are used to indicate the tumors; (**C**) The survival curve of tumor-bearing mice. The survival of mice was recorded since the mice were treated with doxycycline. Moribund mice or mice with a tumor ˃1000 mm^3^ were recorded as dead; and (**D**) Western blotting of Bmi1, apoptosis and cell cycle-associated proteins in the tumor tissues. After the mice were sacrificed, the tumors were collected for western blotting. Data are expressed as the mean ± SD. * *p* < 0.05.

To determine whether the knockdown of Bmi1 can inhibit the tumor growth in fast growing periods *in vivo*, Hep3B cells transfected with Bmi1/pLKO-Tet-On were implanted in nude mice to generate the tumor bearing mice. When the tumors grew to ~100 mm^3^, autoclaved water with 1.5 mg/mL of doxycycline was given to the treatment group mice and regular autoclaved water was continued for control group mice. Ten days after doxycycline treatment, the tumors of treated mice were markedly smaller in comparison with the control group mice (Figure 5A,B). When the tumors of control mice grew to ~200 mm^3^, the tumors of treated mice were only ~100 mm^3^ (Figure 5A,B). The growth curves also showed that Bmi1 KO Hep3B tumors maintained their sizes during the treatments for 10 days, while tumors of the untreated mice kept growing quickly (Figure 5A). Correspondingly, the doxycycline treatment also promoted the survival of tumor-bearing mice. The median survival time of the treated mice was 24 days after tumor formation (100 mm^3^), while for the untreated mice, it was 16 days (Figure 5C). Western blotting of the tumor samples showed that the expression of cyclin D1 and E was decreased, whereas the expression of Bax and caspase-3 was increased (Figure 5D). This clearly indicated that Bmi1 knockdown resulted in cell cycle arrest and apoptosis. Taken together, these data showed that knockdown of Bmi1 expression can inhibit the growth of HCC in fast growing periods *in vivo*.

**Figure 6:**
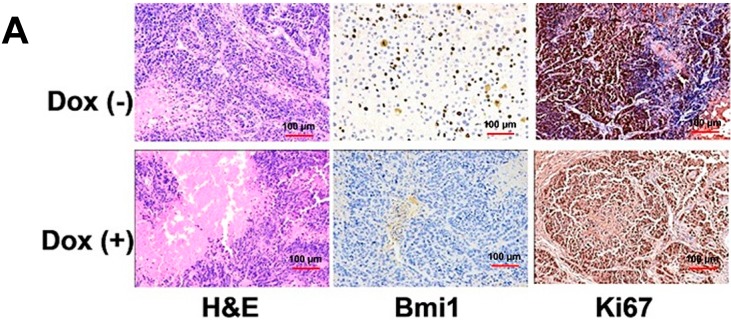

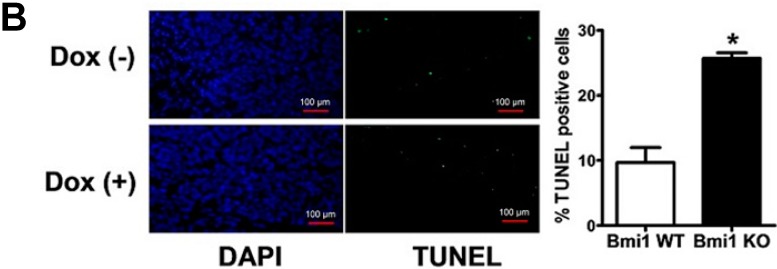
Characterization and apoptosis detection of tumor tissues. (**A**) Immunohistology of tumor tissues in nude mice. Tumor tissues were collected from the moribund mice at the end of the experiment; and (**B**) TUNEL assay in the tumors treated with doxycycline. The percentage of TUNEL-positive cells was determined by counting TUNEL-positive cells and total cells in the same fields (*n* = 3). Data are expressed as the mean ± SD. * *p* < 0.05.

To further ensure that the tumor growth arrest resulted from the Bmi1 knockdown, three moribund mice of each group were sacrificed for immunoblotting and immunohistochemistry after 10 days of doxycycline or regular water treatment. Histologic examination showed that the untreated tumors consisted of frequent trabecular disorganization and exhibited clearly the nuclear expression of Bmi1 (Figure 6A). The Bmi1 staining showed significantly abrogated Bmi1 expression in the doxycycline-treated mice (Figure 6A). Ki67 staining revealed a high level proliferation for WT Hep3B tumors and a relatively low level proliferation of Bmi1 KO tumors (Figure 6A). The TUNEL assay showed that Bmi1 knockdown by doxycycline generated increased apoptotic signals in the treated Hep3B tumor samples (Figure 6B). Combined with the *in vitro* study, these results suggest that the Bmi1 knockdown can inhibit the HCC growth in mice, and the mechanism is mainly due to the induction of cell cycle arrest, while increased apoptosis was observed in tumor tissues.

### 2.5. Discussion

Human HCC is one of the most frequent solid tumors, ranking fifth in incidence and third in mortality worldwide. Screening and validation of the potential therapeutic targets for HCC have long been the mission of fundamental studies. The *Bmi1* gene has been recognized as an oncogene in HCC and many other types of tumors [18,28,29,30]. Using Bmi1 transgenic or knockout mice, several research groups have showed that Bmi1 expression is required for the tumorigenesis of HCC [25,31]. Although some studies showed that repression of Bmi1 can inhibit the growth of HCC cells *in vitro*, more *in vivo* evidence was needed to validate Bmi1 as a therapeutic target for HCC treatment, especially in the late stages. In our present study, we showed that Bmi1 knockdown can inhibit HCC cell growth *in vivo*, both in the initiating stages and progressing stages. Especially, our data indicated that even in the fast growing stages of HCC, repression of Bmi1 can still block the growth of HCC. Our study therefore provides pivotal evidence to support Bmi1 as a target for HCC treatment.

Such kinds of studies have been done by other groups. Similarly, all of the results support Bmi1 as a potential therapeutic target. Ruan *et al.* showed that Bmi1 downregulation inhibits HCC cell growth and tumorsphere formation *in vitro*, blocks cell cycle transition from the G0/G1 to the S phase of HCC cell lines, inhibits tumorigenicity *in vivo* and enhances HCC sensitivity to sorafenib [32]. The results of Zhang *et al.* indicate that the downregulation of the *Bmi1* gene by RNAi can inhibit the proliferation and invasiveness of HCC cells and increases their sensitivity to 5-FU treatment [33]. Wu and colleagues reported that repression of Bmi1 can enhance the sensitivity of osteosarcoma to cisplatin [34]. Wang *et al*. reported that knockdown of Bmi1 also reduces the resistance of ovarian cancer cells to cisplatin [20]. All of these studies imply that the Bmi1 is a potential target for cancer treatments, including HCC.

Although some of these studies used a mouse model to show the efficacy of Bmi1 knockdown on tumor growth inhibition, they failed to provide evidence that repression of Bmi1 in the fast growing stages will be effective. By using a conditional knockdown system, we, for the first time, showed that knockdown of Bmi1 in the fast growing stages, instead of from the onset of tumors, can still inhibit the growth of HCC in a mouse model. Though the xenograft mouse model has many deficiencies, it is still a well-accepted mouse model to evaluate the efficacy of anti-tumor drugs or treatments. However, we are currently evaluating the possibility of using a hydrodynamic injection tumor model for the further evaluation of oncogene targeting treatments.

Yonemitsu *et al*. reported that Bmi1 was overexpressed in 52 tumor tissues (60.5%) of 86 hepatocellular carcinoma surgical specimens, and the cumulative recurrence rate was significantly higher in patients positive for Bmi1 (*p* = 0.039) than in their negative counterparts, as determined by Kaplan–Meier analysis [8]. These data indicate that Bmi1 is associated with the malignant progression of HCC. Our data showed that knockdown of Bmi1 can prolong the survival of tumor-bearing mice from 16 to 24 days, and it seems that eight days is not significant compared to the average life span of nude mice of about 120 days. However, the only approved drug on the market for HCC treatment, sorafenib, can only prolong the median survival time of HCC patients for an average of 3.4 month [9]. Considering that the average life span of a human is about 70 years, an eight-day expansion of the life span for nude mice was a significant improvement, and this equals 56 months for humans. In addition, our other study has shown that co-delivery of Bmi1 siRNA and doxorubicin can significantly prolong the survival of tumor-bearing mice [10], which hints at its possible application together with chemotherapy. Considering the urgent demand for the effective treatment of HCC, the Bmi1 targeting treatment holds promise as one of the future therapeutic options.

Several studies have revealed that Bmi1 maintains tumor growth by inhibiting apoptosis, and knockout of Bmi1 will result in the apoptosis of cancer cells [20,35]. In our study, we observed apoptosis in the xenograft tumors when Bmi1 was knocked down. However, we did not observe the increased apoptosis in the cultured Bmi1 KO Hep3B cells. This is possibly due to the different growing conditions for cultured HCC cells. In cell culture, apoptotic HCC cells will quickly be detached from the culture dish, which makes them difficult to detect. In tumor tissues, however, the tumor microenvironment will help maintain the survival of the apoptotic HCC cells, and thus, apoptotic HCC cells are easier to be detected by TUNEL staining.

Bmi1 exerts its oncogenic function in many types of cancers by silencing the INK4a/ARF (Cdkn2a) tumor suppressor locus encoding the p16 (INK4a) and p14 (ARF) proteins. The p16 inhibits cell cycle progression by disrupting the cyclin D/CDK 4/6 complex, whereas p14 suppresses Mdm2 function and subsequently results in p53 stabilization and subsequent apoptosis. Thus, the tumor-promoting function of the *Bmi1* gene has contributed to the inhibition of cell apoptosis [35]. However, emerging evidence indicates that Bmi1 also functions in an INK4a/ARF independent manner [19,24,25,36,37] or partially dependent manner [18,38] in the tumorigenic context. Our previous study revealed that knockdown of Bmi1 does not increase the mRNA and protein level of p14 and p16 in Huh7, Hep3B and SK-Hep1 cell lines [25]. However, how the Bmi1 KO induced cell cycle arrest was still unresolved. Clearly, the next step in the characterization of molecular mechanisms of Bmi1 is to identify novel targets and/or pathways regulated by Bmi1 during HCC pathogenesis and maintenance.

## 3. Experimental Section

### 3.1. Cell Culture, Short Hairpin RNA Constructs, Lentivirus and Infection

The Huh7 and Hep3B cell lines are from the American Type Culture Collection (ATCC, Manassas, VA, USA). Cells were cultured in Dulbecco’s Modified Eagle’s Medium (Hyclone, Logan, UT, USA) containing 10% fetal bovine serum (Gibco, Gaithersburg, MD, USA), 100 U/mL penicillin (Sigma-Aldrich, St. Louis, MO, USA) and 100 mg/mL streptomycin (Sigma-Aldrich) in 37 °C and 5% CO_2_ incubators. M-plasmocin (Invivogen, San Diego, CA, USA) at a concentration of 2.5 μg/mL was used to prevent possible mycoplasma infection.

ShRNA constructs Bmi1/pLKO.1 (TRCN0000020155, NM_005180.5-693s1c1) used to silence Bmi1 expression were obtained from OpenBiosystems (GE healthcare, New York, NY, USA). Control pLKO.1 (empty vector) or SC/PLKO.1 (with a scrambled sequence) plasmids were obtained from Addgene (Addgene, Madison, MD, USA). The doxycycline inducible Bmi1 shRNA vector was obtained by cloning the Bmi1 shRNA (5'-CCGGCCAGACCACTACTGAATATAACTCGAGTTATATTCAGTAGTGGTCTGGTTTTT-3' and 5'-AATTAAAAACCAGACCACTACTGAATATAACTCGAGTTATATTCAGTAGTGGTCTGG-3') into the AgeI/EcoRI sites of the pLKO-Tet-On vector downstream of the U6 promoter. Then, the vectors were packaged by lentivirus. Lentivirus generated by the biotechnology company (Neuron Biotech, Shanghai, China) was used to infect HCC cells by adding the thawed virus into the cell culture directly. Two days after infection, cells were expanded and selected with 1 μg/mL puromycin for 2 days and harvested for protein or RNA analysis.

### 3.2. RNA Extraction and Real-Time qPCR

Total RNA was extracted using the RNeasy Mini kit (Qiagen, Hilden, Germany). Total RNA of 5 μg was used to synthesize the first strand of cDNA by using SuperScript II RT 200 U/mL (Invitrogen, Carlsbad, CA, USA). The Bmi1 mRNA level was evaluated by real-time PCR on an ABI Prism H7300 (Applied Biosystems, Foster City, CA, USA) with SYBR Green PCR core reagents. The rRNA was applied as the input reference. The primers used in this study are listed below: Bmi1: F, 5'-TGGACTGACAAATGCTGGAGA-3', R, 5'-GAAGATTGGTGGTGGTTACCGCTG-3'; rRNA: F, 5'-CGGCTACCACATCCAAGGAA-3', R, 5'-GCTGGAATTACCGCGGCT-3'.

### 3.3. Protein Lysates and Western Blotting

Protein extracts of cell lines or tissues were prepared by using M-PER mammalian protein extraction reagent (Thermo Fisher, Rockford, IL, USA) plus proteinase inhibitor cocktail (Roche, Indianapolis, IN, USA). Protein concentrations of the lysate were quantified using the BCA protein assay (Beyotime, Beijing, China). Western blotting was done as described elsewhere [39]. Antibodies were prepared as follows: Anti-Bmi1 1:1000 (Millipore, Bedford, MA, USA), anti-cyclin D1 1:1000 (Milipore, Bedford, MA, USA), anti-cyclin E 1:1000 (Abcam, San Francisco, CA, USA), anti-Bcl2 1:1000 (Abcam), anti-Bax 1:1000 (Abcam), anti-caspase 3 1:1000 (Abcam) and anti-α-tubulin 1:1000 (Boster, Wuhan, China).

### 3.4. Cell Proliferation and Cell Cycle Assays

To assay the cell proliferation rate, an equal number of cells was seeded in 6-well plates. After growth, cells were digested by trypsin (Beyotime, Beijing, China) and stained by trypan blue (Beyotime, Beijing, China) at different time points. Then, the cells were counted by a blood counting chamber, and only viable cells were counted. For cell cycle analysis, cells were digested by trypsin and collected with the suspension cells together. After fixation by 70% ethanol, cells were incubated by 0.5 mg/mL RNase and 0.025 mg/mL propidium iodide for 30 min. For each sample, 5 × 10^4^ cells were detected by flow cytometry (CytomicsTM FC 500, Beckman Coulter, CA, USA), and the results were analyzed by using software FlowJo 7.6 (FlowJo, Ashland, OR, USA). BrdU labeling was performed as described in the literature [40].

### 3.5. Analysis of Clonogenicity in Vitro

For colony formation analysis, Hep3B cells were infected by lentivirus for 48 h and then selected by 1 µg/mL puromycin for 48 h. Then, 1000 viable Hep3B cells were plated in 6-well plates and maintained in complete DMEM medium for 1 week. Colonies were fixed with methanol and stained with 0.1% crystal violet in 20% methanol. Triplicate wells were prepared for each transfectant, and the experiment was repeated twice.

### 3.6. Mice and Xenograft Mouse Model

Female BALB/c nude mice (6–8 weeks of age, 16–18 g) were obtained from Huafukang Technology Corporation (Beijing, China), kept in filter-topped cages with standard rodent chow and had water available *ad libitum* and a 12 h light/dark cycle. The experiments were performed according to national regulations and approved by the Animal Experiments Ethical Committee of Huazhong University of Science and Technology. Hep3B cells infected with lentivirus were selected by puromycin and then expanded. For the subcutaneous implantation, 2 × 10^6^, 4 × 10^6^ or 6 × 10^6^ Hep3B cells were injected per nude mouse. After the implantation, tumor growth was observed every other day, and tumor volumes were calculated as *V* = 0.52 × a × b^2^, with “a” representing the long diameter and “b” the short diameter. For the survival record, moribund mice or mice with tumors ˃1000 mm^3^ upon the examination were recorded as dead and sacrificed for histological examination or western blotting.

### 3.7. Histology, Immunohistochemistry and Immunofluorescence

The tumors were collected from the mice after sacrifice and rinsed in 4% cold paraformaldehyde. Fixed tissue samples were embedded in paraffin, and slides were made for Hematoxylin and eosin (H&E) staining and other immunohistochemistry or immunofluorescence. For immunohistochemistry staining, tumor tissue was divided and fixed in 4% paraformaldehyde overnight at 4 °C, then ethanol overnight at 4 °C and processed to be embedded in paraffin blocks. Paraffin slides were dewaxed by xylene, followed by rehydrating through a series of washes with incrementally decreasing percentages of ethanol. Antigen retrieval was performed in 10 mM sodium citrate buffer (pH 6.0) by a placement microwave on high for 10 min, followed by a 20-min cool down at room temperature. After a blocking step with the 5% goat serum and avidin-biotin blocking kit (Vector Laboratories, Burlingame, CA, USA), the slides were incubated with antibodies: anti-Ki67 (Labvision, Fremont, CA, USA) at a 1:150 dilution overnight at 4 °C; anti-Bmi1 (Bethyl) was at a 1:100 dilution overnight at room temperature. Slides were then subjected to 3% hydrogen peroxide for 10 min to quench endogenous peroxidase and, subsequently, the biotin-conjugated secondary antibody 1:400 dilution for 30 min at room temperature. Detection was performed with the ABC-Elite peroxidase kit (Vector Laboratories, Burlingame, CA, USA) by using the DAB substrate kit (DakoCytomation, Carpinteria, CA, USA). For cellular immunofluorescence, cells grown in the cover slip were fixed by 4% cold paraformaldehyde and penetrated by 0.1% Triton X-100 (Thermo, Rockford, IL, USA). Then, cells were incubated with primary antibody and fluorescence conjugated secondary antibody (Alexa Fluor, Invitrogen, Carlsbad, CA, USA). Antibodies and dilutions were used as follows: anti-BrdU 1:1000 (Millipore, Bedford, MA, USA), anti-Cyclin D1 1:1000 (Millipore).

### 3.8. Microscopy

Microscopic examination of cells or tissue section slides was performed with an Olympus SZX12 fluorescence microscope equipped with a digital camera and connected to a PC running MagnaFire 2.0 camera software (Optronics, Goleta, CA, USA). Pictures were taken at equal exposure times for each sample.

### 3.9. Statistical Analysis

All statistical analyses were performed using SPSS13.0 software (SPSS Inc., Chicago, IL, USA). The differences between groups were compared using the Student’s *t*-test, and data were expressed as the mean ± SD. Values of *p* < 0.05 were considered to be significant.

## 4. Conclusions

In the present study, we found that knockdown of the *Bmi1* gene inhibits HCC tumorigenicity and proliferation *in vivo*, both in the onset period and the fast growing period of the neoplasm. We also found that Bmi1 knockdown inhibits the tumor growth by inducing cell cycle arrest and apoptosis. In summary, our results demonstrated that Bmi1 can be used as a potential treatment target for HCC.

## References

[1] Roberts L.R. (2008). Sorafenib in liver cancer—Just the beginning. N. Engl. J. Med..

[2] Shariff M.I., Cox I.J., Gomaa A.I., Khan S.A., Gedroyc W., Taylor-Robinson S.D. (2009). Hepatocellular carcinoma: Current trends in worldwide epidemiology, risk factors, diagnosis and therapeutics. Expert Rev. Gastroenterol. Hepatol..

[3] Matsushita E., Unoura M., Kaneko S., Kobayashi K. (1995). Risk factors for development of hepatocellular carcinoma in patients with liver cirrhosis associated with hepatitis C virus. Nihon Rinsho.

[4] Zaman S.N., Melia W.M., Johnson R.D., Portmann B.C., Johnson P.J., Williams R. (1985). Risk factors in development of hepatocellular carcinoma in cirrhosis: Prospective study of 613 patients. Lancet.

[5] Feo F., Pascale R.M., Simile M.M., de Miglio M.R., Muroni M.R., Calvisi D. (2000). Genetic alterations in liver carcinogenesis: Implications for new preventive and therapeutic strategies. Crit. Rev. Oncog..

[6] Thorgeirsson S.S., Grisham J.W. (2002). Molecular pathogenesis of human hepatocellular carcinoma. Nat. Genet..

[7] Whittaker S., Marais R., Zhu A.X. (2010). The role of signaling pathways in the development and treatment of hepatocellular carcinoma. Oncogene.

[8] Forner A., Llovet J.M., Bruix J. (2012). Hepatocellular carcinoma. Lancet.

[9] Levy L., Renard C.A., Wei Y., Buendia M.A. (2002). Genetic alterations and oncogenic pathways in hepatocellular carcinoma. Ann. N. Y. Acad. Sci..

[10] Jacobs J.J., Kieboom K., Marino S., de Pinho R.A., van Lohuizen M. (1999). The oncogene and Polycomb-group gene *Bmi1* regulates cell proliferation and senescence through the *INK4a* locus. Nature.

[11] Park I.K., Qian D., Kiel M., Becker M.W., Pihalja M., Weissman I.L., Morrison S.J., Clarke M.F. (2003). *Bmi1* is required for maintenance of adult self-renewing haematopoietic stem cells. Nature.

[12] Molofsky A.V., Pardal R., Iwashita T., Park I.K., Clarke M.F., Morrison S.J. (2003). *Bmi1* dependence distinguishes neural stem cell self-renewal from progenitor proliferation. Nature.

[13] Park I.K., Morrison S.J., Clarke M.F. (2004). *Bmi1*, stem cells, and senescence regulation. J. Clin. Investig..

[14] Jacobs J.J., Scheijen B., Voncken J.W., Kieboom K., Berns A., van Lohuizen M. (1999). *Bmi1* collaborates with c-Myc in tumorigenesis by inhibiting c-Myc-induced apoptosis via INK4a/ARF. Genes Dev..

[15] Pietersen A.M., Horlings H.M., Hauptmann M., Langerod A., Ajouaou A., Cornelissen-Steijger P., Wessels L.F., Jonkers J., van de Vijver M.J., van Lohuizen M. (2008). EZH2 and Bmi1 inversely correlate with prognosis and TP53 mutation in breast cancer. Breast Cancer Res..

[16] Dhawan S., Tschen S.I., Bhushan A. (2009). Bmi1 regulates the *INK4a/ARF* locus to control pancreatic beta-cell proliferation. Genes Dev..

[17] Becker M., Korn C., Sienerth A.R., Voswinckel R., Luetkenhaus K., Ceteci F., Rapp U.R. (2009). Polycomb group protein *Bmi1* is required for growth of RAF driven non-small-cell lung cancer. PLoS One.

[18] Dovey J.S., Zacharek S.J., Kim C.F., Lees J.A. (2008). Bmi1 is critical for lung tumorigenesis and bronchioalveolar stem cell expansion. Proc. Natl. Acad. Sci. USA.

[19] Bruggeman S.W., Hulsman D., Tanger E., Buckle T., Blom M., Zevenhoven J., van Tellingen O., van Lohuizen M. (2007). Bmi1 controls tumor development in an INK4a/ARF-independent manner in a mouse model for glioma. Cancer Cell.

[20] Wang E., Bhattacharyya S., Szabolcs A., Rodriguez-Aguayo C., Jennings N.B., Lopez-Berestein G., Mukherjee P., Sood A.K., Bhattacharya R. (2011). Enhancing chemotherapy response with Bmi1 silencing in ovarian cancer. PLoS One.

[21] Qin Z.K., Yang J.A., Ye Y.L., Zhang X., Xu L.H., Zhou F.J., Han H., Liu Z.W., Song L.B., Zeng M.S. (2009). Expression of Bmi1 is a prognostic marker in bladder cancer. BMC Cancer.

[22] Song L.B., Zeng M.S., Liao W.T., Zhang L., Mo H.Y., Liu W.L., Shao J.Y., Wu Q.L., Li M.Z., Xia Y.F. (2006). Bmi1 is a novel molecular marker of nasopharyngeal carcinoma progression and immortalizes primary human nasopharyngeal epithelial cells. Cancer Res.

[23] Mohty M., Yong A.S., Szydlo R.M., Apperley J.F., Melo J.V. (2007). The polycomb group *Bmi1* gene is a molecular marker for predicting prognosis of chronic myeloid leukemia. Blood.

[24] Chiba T., Miyagi S., Saraya A., Aoki R., Seki A., Morita Y., Yonemitsu Y., Yokosuka O., Taniguchi H., Nakauchi H. (2008). The polycomb gene product Bmi1 contributes to the maintenance of tumor-initiating side population cells in hepatocellular carcinoma. Cancer Res..

[25] Xu C.R., Lee S., Ho C., Bommi P., Huang S.A., Cheung S.T., Dimri G.P., Chen X. (2009). Bmi1 functions as an oncogene independent of INK4a/ARF repression in hepatic carcinogenesis. Mol. Cancer Res..

[26] Chiba T., Seki A., Aoki R., Ichikawa H., Negishi M., Miyagi S., Oguro H., Saraya A., Kamiya A., Nakauchi H. (2010). *Bmi1* promotes hepatic stem cell expansion and tumorigenicity in both *INK4a/ARF*-dependent and -independent manners in mice. Hepatology.

[27] Sparmann A., van Lohuizen M. (2006). Polycomb silencers control cell fate, development and cancer. Nat. Rev. Cancer.

[28] Douglas D., Hsu J.H., Hung L., Cooper A., Abdueva D., van Doorninck J., Peng G., Shimada H., Triche T.J., Lawlor E.R. (2008). *Bmi1* promotes ewing sarcoma tumorigenicity independent of *CDKN2A* repression. Cancer Res..

[29] Wiederschain D., Chen L., Johnson B., Bettano K., Jackson D., Taraszka J., Wang Y.K., Jones M.D., Morrissey M., Deeds J. (2007). Contribution of polycomb homologues Bmi1 and Mel-18 to medulloblastoma pathogenesis. Mol. Cell Biol..

[30] Fan C., He L., Kapoor A., Gillis A., Rybak A.P., Cutz J.C., Tang D. (2008). Bmi1 promotes prostate tumorigenesis via inhibiting p16^INK4a^ and p14^ARF^ expression. Biochim. Biophys. Acta.

[31] Chiba T., Zheng Y.W., Kita K., Yokosuka O., Saisho H., Onodera M., Miyoshi H., Nakano M., Zen Y., Nakanuma Y. (2007). Enhanced self-renewal capability in hepatic stem/progenitor cells drives cancer initiation. Gastroenterology.

[32] Ruan Z.P., Xu R., Lv Y., Tian T., Wang W.J., Guo H., Nan K.J. (2013). Bmi1 knockdown inhibits hepatocarcinogenesis. Int. J. Oncol..

[33] Zhang R., Xu L.B., Yue X.J., Yu X.H., Wang J., Liu C. (2013). *Bmi1* gene silencing inhibits the proliferation and invasiveness of human hepatocellular carcinoma cells and increases their sensitivity to 5-fluorouracil. Oncol. Rep..

[34] Wu Z., Min L., Chen D., Hao D., Duan Y., Qiu G., Wang Y. (2011). Overexpression of *Bmi1* promotes cell growth and resistance to cisplatin treatment in osteosarcoma. PLoS One.

[35] Jagani Z., Wiederschain D., Loo A., He D., Mosher R., Fordjour P., Monahan J., Morrissey M., Yao Y.M., Lengauer C. (2010). The Polycomb group protein Bmi1 is essential for the growth of multiple myeloma cells. Cancer Res..

[36] Datta S., Hoenerhoff M.J., Bommi P., Sainger R., Guo W.J., Dimri M., Band H., Band V., Green J.E., Dimri G.P. (2007). Bmi1 cooperates with H-Ras to transform human mammary epithelial cells via dysregulation of multiple growth-regulatory pathways. Cancer Res..

[37] Hoenerhoff M.J., Chu I., Barkan D., Liu Z.Y., Datta S., Dimri G.P., Green J.E. (2009). Bmi1 cooperates with H-Ras to induce an aggressive breast cancer phenotype with brain metastases. Oncogene.

[38] Lessard J., Sauvageau G. (2003). Bmi1 determines the proliferative capacity of normal and leukaemic stem cells. Nature.

[39] Tward A.D., Jones K.D., Yant S., Cheung S.T., Fan S.T., Chen X., Kay M.A., Wang R., Bishop J.M. (2007). Distinct pathways of genomic progression to benign and malignant tumors of the liver. Proc. Natl. Acad. Sci. USA.

[40] Wojtowicz J.M., Kee N. (2006). BrdU assay for neurogenesis in rodents. Nat. Protoc..

